# Adult-onset STING-associated vasculopathy

**DOI:** 10.70962/jhi.20250235

**Published:** 2026-05-11

**Authors:** Thomas R. Riley, Jonathan J. Kotzin, Debby J. Park, Shubhasree Banerjee, Asako Takanohashi, Adeline Vanderver, Penn Medicine Biobank, Daniel J. Rader, Marylyn D. Ritchie, JoEllen Weaver, Nawar Naseer, Giorgio Sirugo, Afiya Poindexter, Yi-An Ko, Kyle P. Nerz, Meghan Livingstone, Fred Vadivieso, Stephanie DerOhannessian, Teo Tran, Julia Stephanowski, Salma Santos, Ned Haubein, Joseph Dunn, Anurag Verma, Colleen Morse Kripke, Marjorie Risman, Renae Judy, Colin Wollack, Shefali S. Verma, Scott Damrauer, Yuki Bradford, Scott Dudek, Theodore Drivas, Joshua F. Baker, Amanda V. Finck, Jonathan J. Miner

**Affiliations:** 1 Division of Rheumatology, Department of Medicine, University of Pennsylvania Perelman School of Medicine, Philadelphia, PA, USA; 2 Division of Neurology, Department of Pediatrics, Children’s Hospital of Philadelphia, Philadelphia, PA, USA; 3 Department of Neurology, University of Pennsylvania Perelman School of Medicine, Philadelphia, PA, USA; 4 Department of Microbiology, University of Pennsylvania Perelman School of Medicine, Philadelphia, PA, USA; 5 Institute for Immunology and Immune Health, University of Pennsylvania Perelman School of Medicine, Philadelphia, PA, USA; 6 RVCL Research Center, University of Pennsylvania Perelman School of Medicine, Philadelphia, PA, USA; 7 Colton Center for Autoimmunity, University of Pennsylvania Perelman School of Medicine, Philadelphia, PA, USA

## Abstract

Genetic contributions to systemic autoimmunity are often considered more significant in children than in adults. As such, genetic evaluation may be more frequently pursued in pediatric rheumatology patients. Motivated by the discovery of a STING-associated vasculopathy with onset in infancy (SAVI) mutation in a patient with adult-onset relapsing polychondritis and systemic lupus erythematosus, we hypothesized that STING gain-of-function mutations might underlie a broader spectrum of autoimmune disease in adults. We systematically screened 43,731 exomes from the Penn Medicine Biobank, revealing five additional unrelated adults with SAVI-associated STING gain-of-function mutations, including several patients with clinical features of SAVI, as well as asymptomatic individuals with type I IFN signatures. We propose the term adult-onset STING-associated vasculopathy (AO-SAVI) to describe these patients. Our findings challenge the conventional symptom-driven diagnostic paradigm, revealing that parallel molecular classification can uncover shared mechanisms and genetic etiologies across seemingly distinct diseases.

## Teaser

Riley, Kotzin et al. screened 43,731 adult exomes and identified six individuals with STING gain-of-function mutations that cause pediatric SAVI. These adults presented with phenotypes ranging from lupus and polychondritis to clinical non-penetrance, none previously diagnosed with SAVI. This establishes AO-SAVI, suggesting that monogenic inborn errors of immunity may be underrecognized in adults.

## Introduction

Rheumatological diseases are traditionally classified based on clinical symptoms and serological markers, creating diagnostic categories such as systemic lupus erythematosus (SLE), rheumatoid arthritis, and systemic vasculitis. However, this symptom-based approach may obscure shared molecular etiologies, particularly in patients with monogenic inborn errors of immunity. Over 490 such disorders have been identified, predominantly in pediatric populations presenting with autoinflammation, autoimmunity, or immunodeficiency (1, 2, 3). The current paradigm is that pediatric-onset autoimmune diseases are more likely to be driven by genetic factors. Here, we challenge this paradigm by identifying adults with disease-causing mutations typically linked to a pediatric autoimmune disease.

Interferonopathies are a class of monogenic autoinflammatory diseases characterized by constitutive activation of type I interferons (IFNs) and increased expression of IFN-stimulated genes (ISGs), typically presenting in childhood (1, 4, 5, 6). A classic interferonopathy, Aicardi-Goutières Syndrome (AGS), can arise from TREX1 loss-of-function mutations leading to accumulation of cytosolic DNA, activation of the DNA sensor cyclic GMP-AMP synthase, and sustained downstream signaling through STING (7, 8). Patients with AGS develop severe neuroinflammation that ultimately results in disability and shortened lifespan. In contrast, gain-of-function mutations in STING cause a clinically distinct syndrome, STING-associated vasculopathy with onset in infancy (SAVI), which is also characterized by increased ISG expression, but instead results in pulmonary, cutaneous, and systemic inflammation (9, 10). SAVI is almost universally regarded as a pediatric disease, with only very rare adult-onset cases previously reported (5, 10, 11, 12, 13, 14). Despite the established role of STING in activating the type I IFN response, it is less clear whether cytokine or ISG profiling could function as a first-line screening tool for SAVI patients prior to genetic testing.

Advances in affordable exome sequencing now enable a gene-first approach to uncover disease-causing mutations and redefine diagnosis boundaries. We identified a SAVI-associated mutation in a patient with a long-standing history of adult-onset relapsing polychondritis and SLE. This led us to hypothesize that STING gain-of-function mutations, classically linked to SAVI, underlie a spectrum of adult-onset autoimmune diseases. We proceeded to screen 43,731 adult exomes from the Penn Medicine Biobank (PMBB) and identified six individuals with STING1 gain-of-function mutations known to cause SAVI in children, revealing a prevalence of 1.1 per 10,000 individuals in our adult health system. None had been previously diagnosed with SAVI, and several exhibited clinical features of the syndrome alongside more common autoimmune disease diagnoses. Others with STING gain-of-function mutations showed clinical non-penetrance. Notably, these patients would not have been easily identified based on immunological parameters, including type I IFN levels. Our results highlight the phenotypic variability of SAVI in adults, underscoring the value of genetic testing beyond childhood disease.

## Results

**Figure 1.**
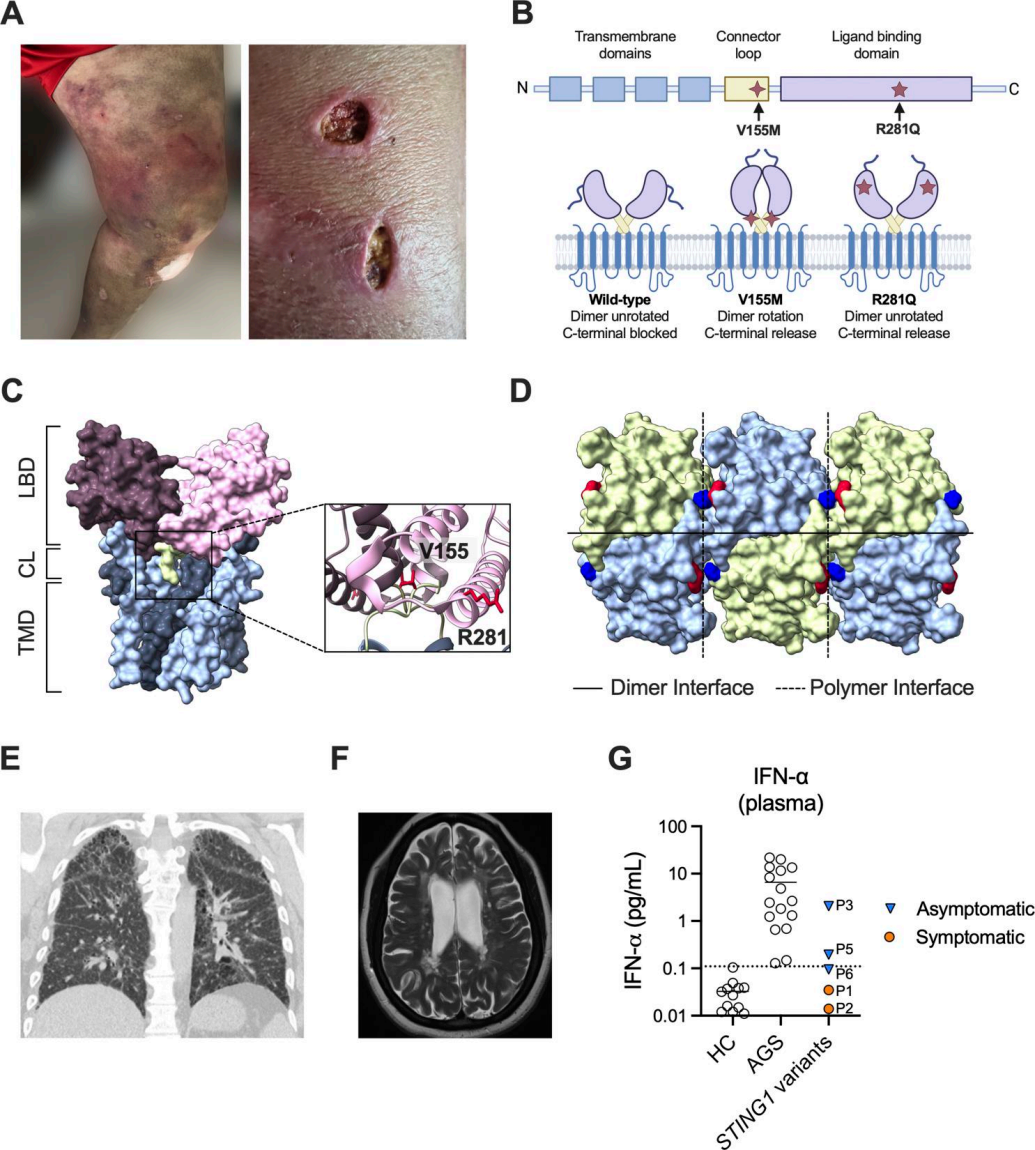
**Clinical and structural basis of AO-SAVI caused by STING gain-of-function mutations. (A)** Representative images of cutaneous vasculopathy in a patient with STING R281Q, showing purpuric rashes (left) and ulcerated skin lesions with biopsies revealing thrombotic vasculopathy (right). **(B)** Top: Schematic of the linear domain architecture of STING with locations of V155M (connector loop) and R281Q (polymeric interface) mutations. Bottom: Schematic of conformational states; wild-type (dimer unrotated, C-terminal blocked), V155M (dimer rotation, C-terminal release), and R281Q (dimer unrotated, C-terminal release). Created with BioRender.com, released under a CC-BY 4.0 license. **(C)** Human WT STING dimer in the apo state (PBD: 6NT5). On the left, the N-terminal transmembrane domain (TMD) (bottom, blue), connector loop (CL) (middle, green), and ligand-binding domain (LBD) (top, purple) are indicated. Insets show V155 and R281 position. **(D)** Crystal lattice of six human STING (H232) monomers bound to cGAMP (PBD: 4LOH), denoting polymeric interface (black dotted line) and the interaction between residue R281 (red) and D301 (blue). Crystal packing patterns are thought to mimic native STING polymerization (17). **(E)** Chest computed tomography image demonstrating interstitial lung disease in a symptomatic patient with the STING V155M mutant (patient 4). **(F)** Brain MRI (T2-weighted image) showing demyelinating CNS inflammatory lesions in a symptomatic patient with the STING V155M mutation (patient 5). **(G)** Plasma IFN-α concentrations measured by digital ELISA in healthy control individuals (HC, *n* = 12), AGS (*n* = 16), and individuals with *STING1* variants identified through the PMBB (*n* = 5). Each point represents a single patient, and the dotted line indicates three times the standard deviation above the HC mean. Blue triangles denote asymptomatic individuals; orange circles denote symptomatic patients on immunosuppressive therapy. cGAMP, cyclic GMP-AMP.

We identified a case of adult-onset STING-associated vasculopathy (AO-SAVI) in a 42-year-old woman, who presented with a 23-year history of refractory SLE and relapsing polychondritis, beginning at age 19. SAVI is conventionally thought to occur primarily during infancy or early childhood, and unlike most patients with SAVI, she did not have interstitial lung disease. The most prominent clinical manifestations were a photosensitive rash, inflammatory arthritis, and serositis. Laboratory evaluation revealed high-titer antinuclear antibodies (1:10,240), hypocomplementemia, lymphopenia, and anti–double-stranded DNA antibodies, consistent with SLE. At age 20, she developed episodic ear and nose inflammation, ultimately leading to a second diagnosis of relapsing polychondritis. Over the course of two decades, she developed tracheal inflammation, deep vein thrombosis with positive anti-cardiolipin antibodies, purpuric rashes, and ulcerated skin lesions with biopsies revealing thrombotic vasculopathy (Fig. 1 A). Despite aggressive treatment with hydroxychloroquine, methotrexate, dapsone, azathioprine, mycophenolate mofetil, and cyclophosphamide, she remained corticosteroid dependent. Anakinra provided only partial relief of her joint pains and rashes, and disease control was only maintained with a significant dose of prednisone. Her refractory disease course was further complicated by recurrent infections—otitis media, sinusitis, pneumonia, and cutaneous *Mycobacteroides chelonae*—ultimately culminating in necrotizing fasciitis of the leg, which necessitated an above-the-knee amputation. Clinical exome sequencing revealed a pathogenic STING R281Q (c.842 G>A) mutation (15), which lies at the STING polymer interface (Fig. 1, B–D) and is well-described in pediatric-onset SAVI. This case of adult-onset disease, which we propose be termed AO-SAVI, prompted us to investigate the prevalence of *STING1* variants in a larger pool of adult patients. To address this question, we leveraged the PMBB, a diverse hospital-based biorepository with 43,731 patient exomes and serum samples linked to electronic health records (Table 1). Screening these exomes for STING gain-of-function variants typically observed in children with SAVI (Table S1) identified five additional patients. Among the variants detected were V155M, located in the connector loop, which promotes rotation of the ligand-binding domain (16), and R281Q, at the polymeric interface, which drives constitutive STING polymerization (Table 2) (17). Thus, within the PMBB, the prevalence of STING gain-of-function variants is 1.1 per 10,000 (95% confidence interval [CI]: 0.3–2.7 per 10,000).

**Table 1.** Patient characteristics in the PMBB

Total	43,884
**Gender**	​
Female	21,965 (50.1%)
Male	21,918 (49.9%)
Other	1 (<1%)
**Age range (years)**	18–103
**Age groups**	​
18–29	4,302 (9.8%)
30–39	5,406 (12.3%)
40–49	5,688 (13.0%)
50–59	9,519 (21.7%)
60–69	10,839 (24.7%)
70–79	5,941 (13.5%)
80+	2,189 (5.0%)
**Electronic health record (EHR)–reported race**	​
African American	10,815 (24.6%)
White	29,329 (66.8%)
Asian	979 (2.2%)
Other	1,372 (3.1%)
Unknown	1,761 (4.0%)
**EHR-reported ethnicity**	​
Hispanic	1,112 (2.5%)
Non-Hispanic	42,425 (96.7%)
Unknown	347 (0.8%)

**Table 2.** Variable penetrance of adults carrying STING gain-of-function mutations

Patient	1	2	3	4	5	6
STING variant	R281Q	R281Q	R281Q	V155M	V155M	V155M
STING AQ	Unknown	AQ/Q	AQ/WT	WT/WT	AQ/AQ	AQ/WT
Age of onset	19	NA	NA	30	30	NA
Age at last follow-up	44	59	67	45	47	24
Cutaneous vasculopathy	Yes	No	No	No	No	No
Interstitial lung disease	No	No	No	Yes	No	No
Inflammatory arthritis	Yes	No	No	Yes	No	No
CNS inflammation	No	No	No	No	Yes	No

Clinical and genetic characteristics of the six identified individuals with pathogenic *STING1* variants. NA, not available.

Of the newly identified patients, one harbored a connector loop STING mutation and presented with classic SAVI features, including interstitial lung disease, inflammatory arthritis, positive rheumatoid factor, and antinuclear antibodies. This patient’s disease also began in adulthood, starting at age 30, and their interstitial lung disease (ILD) was stabilized on azathioprine following glucocorticoid treatment (Fig. 1 E). Similarly, another patient with STING V155M presented at age 30 with demyelinating disease consistent with multiple sclerosis (MS), which was refractory to dimethyl fumarate and considered responsive to B cell depletion with ocrelizumab (Fig. 1 F). Brain lesions and neuroinflammation have been described in humans and mice with SAVI-associated STING mutations, supporting the link between chronic STING activation and central nervous system (CNS) pathology (1, 5, 18, 19). The remaining three individuals (patients 2, 3, and 6; two with STING R281Q and one with STING V155M) were asymptomatic, even in late adulthood (ages at last follow-up: 67, 59, and 24), suggesting incomplete penetrance (Table 2 and Table S2). One of the asymptomatic individuals with STING R281Q had HIV controlled with antiretroviral therapy (ART).

To explore variable penetrance, we examined whether these individuals have mitigating *STING1* variants, such as the AQ haplotype (G230A-R293Q), known to reduce SAVI pathology in mice (20). All three asymptomatic individuals were heterozygous for the AQ haplotype, while the symptomatic patient with MS was homozygous for AQ (Table 2). Plasma IFN-α levels were elevated in untreated asymptomatic individuals compared to both healthy controls and symptomatic patients on immunosuppression. In the asymptomatic cases, the increase in plasma IFN-α was similar to that of AGS patients (Fig. 1 G), the prototypical interferonopathy associated with chronic STING activation (1, 4). Immunosuppressive therapy may explain absence of an IFN signature in the symptomatic AO-SAVI patients, though we cannot exclude that low levels of type I IFN may reflect variability in plasma processing times. Although these individuals could not be recalled for additional sample collection and clinical phenotyping, our findings demonstrate marked variability in penetrance and age of disease onset among carriers of STING gain-of-function variants.

## Discussion

Within a single academic medical center, we identified six individuals with STING gain-of-function mutations known to cause childhood-onset SAVI. These patients’ adult-onset disease phenotypes ranged from classic SAVI features such as skin ulcerations and ILD to complete clinical non-penetrance well into adulthood. These findings demonstrate that a parallel, molecularly defined classification system can unify seemingly disparate adult presentations to enable more precise diagnosis and treatment.

SAVI was initially defined as infantile-onset vasculopathy, and all of the published cases of adult-onset disease retain core pediatric disease features (5, 10, 11, 12, 13, 14), with testing of adults usually prompted by a familial pediatric case (5, 21). By interrogating exomes across our health system, our study more than doubles the number of AO-SAVI cases in the literature and expands the spectrum of illness from clinical non-penetrance to disease phenotypes mimicking common autoimmune syndromes (9, 21).

While brain lesions have been reported in humans and mice with SAVI mutations (1, 12), the CNS disease of patient 5 may not be directly related to her STING mutation. Given the high prevalence of MS in the United States (22) and the favorable response of this patient to B cell depletion, the contribution of STING gain-of-function to this particular case remains uncertain. In our cohort, three of six carriers were asymptomatic. These patients were heterozygous for the AQ haplotype, which partially ameliorates SAVI pathology in animal models (20). Beyond AQ haplotypes, other mechanisms of incomplete penetrance may include somatic mosaicism, monoallelic expression, and other genetic modifiers and environmental triggers (15, 23).

Type I IFN signaling is a hallmark of SAVI (1), yet our plasma IFN-α measurements suggest that circulating type I IFN is an imperfect surrogate for the diagnosis. While caveats to our testing parameters exist, only two of five individuals had clear elevations in their plasma IFN-α levels, and both of these individuals were asymptomatic. The caveats of these cytokine assays can be broadly categorized as technical and biological in nature. Technically, SAVI samples were processed between 1 and 4 h after collection, which may have impacted cytokine levels in these plasma samples. However, delayed plasma processing is unlikely to explain increased IFN-α in the two asymptomatic individuals. Regarding biologic factors, both symptomatic patients were receiving immunomodulatory therapy, and it is possible that these therapies reduced the plasma IFN-α levels either directly or indirectly. Further, exposures such as viral infections may elevate IFN-α levels, which might explain an IFN signature at a single time point in the asymptomatic patients. Importantly, asymptomatic patient 3 was known to have an ART-suppressed HIV infection, and this individual was also the only asymptomatic patient without a clear increase in plasma IFN-α. Notably, murine models of SAVI demonstrate that clinical disease depends more on type II IFN than on type I IFN (24), and partial rescue by the AQ haplotype occurs independently of changes in IFN signaling (20). Thus, the role of type I IFN and genetic modifiers in SAVI are topics of ongoing research.

We recognize several limitations of our work. Although the PMBB is a diverse cohort reflective of the regional health network, the variant frequencies may differ from other populations and community-based biobanks. Nonetheless, exome identity by descent analysis confirmed that these individuals were unrelated and did not represent familial clusters of these variants. An additional caveat is that phenotypic assignment relied on documentation from routine clinical encounters, where subtle manifestations may not have been captured. Finally, we were unable to recall patients for additional pedigree analysis and peripheral blood mononuclear cell collection. This would have allowed quantitation of ISG expression and immunological parameters that might provide insight into mechanisms explaining diverse clinical phenotypes and non-penetrance.

Our findings challenge the conventional symptom-based diagnostic framework, which relies heavily on pattern recognition and routine immunophenotyping, such as serologies, cytokine panels, and cellular assays, which often fail to define core mechanisms of immunopathology. The prevalence of STING mutations within the Penn Medicine Health System (1.1 per 10,000 adults) suggests that monogenic inborn errors of immunity are likely underrecognized contributors to adult disease. Similar approaches should be considered for other autoinflammatory conditions, such as COPA syndrome or familial Mediterranean fever, which can masquerade as more common adult autoimmune conditions. A more routine use of genetic diagnosis has the potential to uncover hidden inborn errors of immunity, refine prognosis, and guide clinical monitoring. Ultimately, the hope is that finding these patients will enable future testing of true precision therapies, such as small molecule inhibitors and proteolysis-targeting chimeras, from which these patients stand to benefit (25, 26).

## Materials and methods

### Study participants and exome sequencing

The source population for this study was the PMBB, a large hospital-based biorepository linked to electronic health records associated with the University of Pennsylvania and Penn Medicine health system (27, 28). 43,731 individuals in PMBB had undergone whole-exome sequencing. DNA was extracted from stored buffy coats. Exome sequences were generated by the Regeneron Genetics Center (Tarrytown, NY, USA) using a high-throughput fully automated approach. DNA libraries were prepared using enzymatic shearing to 100 ng of genomic DNA with a mean fragment size of 200 base pairs with a custom NEBNext Ultra II FS DNA library kit (New England Biolabs). Unique asymmetric barcodes, 10 base pair in length, were added during library amplification using KAPA HiFi polymerase (KAPA Biosystems), enabling multiplexed exome capture and sequencing. Samples were pooled in equal amounts for overnight exome capture using a modified version of Integrated DNA Technology’s xGen probe library, with supplemental probes used to cover regions poorly captured by standard xGen probes (NimbleGen VCRome). Captured fragments were bound to streptavidin-coupled DNA beads (Thermo Fisher Scientific) and washed using xGen Hybridization and Wash Kit (Integrated DNA Technology). DNA was PCR amplified with KAPA HiFi and quantified by quantitative PCR using the KAPA Library Quantification Kit (KAPA Biosystems). Pooled libraries were sequenced with 75 base pair paired-end reads and two 10 base pair index reads using the Illumina NovaSeq 6000 platform using S4 flow cells. As previously described, exomes were mapped to GRCh38 following the original quality functional equivalent protocol and additional quality control steps were performed to filter data based on individual-level missingness and read depth (27, 29, 30, 31). A manually curated list of pathogenic and likely pathogenic gain-of-function variants in STING1 was generated from ClinVar and literature (Table S1) (6, 13, 18, 21, 32, 33, 34). This list was used to screen PMBB whole exomes to identify participants. Common genetic variants associated with reduced *STING1* activity were also extracted, including *STING1* R232H and the *STING1* AQ haplotype comprised of G230A and R293Q (Table S1) (15, 20, 23, 27). Patient manifestations and age of onset were extracted from review of electronic health records by manual chart review by a rheumatologist. Identity by descent was performed using genomic data for the identified cases to verify relatedness. Study data were collected and managed using REDCap electronic data capture tools hosted at the University of Pennsylvania (35, 36). Participants in PMBB provided informed consent under the University of Pennsylvania Institutional Review Board protocol 813913. Analysis and data extraction were performed using STATA (v18), VCFtools, and PLINK (v1.9) (37, 38).

### Plasma samples

All plasma samples were processed according to standard laboratory procedures and stored at −80°C until analysis. For the PMBB cohort, EDTA-treated whole blood samples were processed within 1–4 h of collection and centrifuged at 1,500 rcf for 15 min at room temperature prior to plasma isolation and storage. For the healthy control cohort, EDTA-treated whole blood samples were processed within 1 h of collection and centrifuged at 1,200 rcf for 15 min at room temperature prior to plasma isolation and storage. For the AGS cohort, heparin- or EDTA-treated whole blood samples were processed within 48 h of collection and centrifuged at 2,000 rcf for 5 min at room temperature prior to plasma isolation and storage. Plasma samples for the five identified PMBB participants were retrieved from PMBB under Penn Institutional Review Board (IRB) protocol 855701 (Genetic Mutations as Adult Immune Disease Drivers). Deidentified healthy control plasma samples of similar sex and age distribution to the identified PMBB participants were collected under Penn IRB protocol 845061 (circulating T cell subsets in healthy donors). AGS plasma samples were collected by the Vanderver lab from subjects with mutations known to cause AGS under the Children’s Hospital of Philadelphia IRB protocol 14-011236 (Myelin Disorders Bioregistry Project).

### Simoa IFN-α digital ELISA testing

The Simoa IFN-α MS advantage PLUS kit was purchased from Quanterix and run by the Human Immunology Core at the University of Pennsylvania on a Simoa HD-X machine according to the manufacturer’s recommendations. Simoa HD-X software was used to interpolate sample concentrations based upon the standard curve. One AGS sample was above the linear phase of the standard curve, and the value was extrapolated. One AGS sample was above all values of the standard curve, and the concentration was unable to be calculated.

### Online supplemental material

Supplemental material for this article includes information on the contributors to the PMBB. Supplemental tables are provided summarizing the STING gain-of-function mutations screened in the analysis (Table S1) and the clinical and molecular features of individuals identified with STING gain-of-function mutations (Table S2).

## Supplementary Material

Table S1 shows STING1 variants tested in this study.

Table S2 shows patient characteristics with reported pathogenic *STING1* variants.

Table S3 shows the Penn Medicine Biobank members and contributions.

## Data Availability

Due to research participant privacy protections, individual-level exome data are not made publicly available. Requests for access to individual-level data relevant to this manuscript can be made by contacting the corresponding authors. Cytokine data are also available upon request.
