# Supplementary material for: Adult-onset STING-associated vasculopathy
Source: J Hum Immun. 2026 May 11;2(4):e20250235. doi: 10.70962/jhi.20250235 (PMC13159526; doi:10.70962/jhi.20250235)
Supplement: Table S1 — shows STING1 variants tested in this study. [file jhi_20250235_tables1.docx]

| **Nucleotide** | **Nucleotide change** | **Amino acid change** | **Inheritance pattern** | **Chromosome** | **Position (GRCh38)** | **Reference** |
| --- | --- | --- | --- | --- | --- | --- |
| **Common variants associated with reduced STING function** | | | |  |  |  |
| 214 | C>A | H72N | AD | 5 | 139481491 | (A) |
| 439 | G>A | V147M | AD | 5 | 139480871 | (B) |
| 439 | G>C | V147L | AD | 5 | 139480871 | (C) |
| 457 | T>A | F153I | AD | 5 | 139480853 | (D) |
| 457 | T>G | F153V | AD | 5 | 139480853 | (A) |
| 461 | A>G | N154S | AD | 5 | 139480849 | (C) |
| 463 | G>A | V155M | AD | 5 | 139480847 | (C) |
| 473 | G>C | G158A | AD | 5 | 139480837 | (A) |
| 497 | G>A | G166E | AD | 5 | 139480813 | (E) |
| 616 | T>G | C206G | AD | 5 | 139478413 | (F) |
| 617 | G>A | C206Y | AD | 5 | 139478412 | (G) |
| 620 | G>A | G207E | AD | 5 | 139478409 | (H) |
| 835 | T>C | F279L | AD | 5 | 139477440 | (I) |
| 841 | C>T | R281W | AR | 5 | 139477434 | (J) |
| 842 | G>A | R281Q | AD | 5 | 139477433 | (G) |
| 850 | A>G | R284G | AD | 5 | 139477425 | (G) |
| 852 | G>T | R284S | AD | 5 | 139477423 | (K) |
| **Common variants associated with reduced STING function** | | | |  |  |  |
| 212 | G>A | R71H | - | 5 | 139481493 | (L) |
| 689 | G>C | G230A | - | 5 | 139478340 | (L) |
| 695 | G>A | R232H | - | 5 | 139478334 | (L-M) |
| 878 | G>A | R293Q | - | 5 | 139477397 | (L) |

**Supplemental Table 1.** STING1 variants tested for in this study. References for the variants include (A) Lin et al. 2021, (B) Munoz et al. 2015, (C) Liu et al. 2014, (D) ClinVar, unpublished, (E) König et al. 2017, (F) Zampeli et al. 2017, (G) (Melki et al 2017, (H) Keskitalo et al. 2019, (I) Seo et al. 2017, (J) Lin et al. 2020, (K) Konno et al. 2018, (L) Jin et al. 2011, (M) Yi et al. 2013.

**Full list of references from the table**

1. Lin B, Torreggiani S, Kahle D, et al. Case report: novel SAVI-causing variants in STING1 expand the clinical disease spectrum and suggest a refined model of STING activation. Front Immunol. 2021;12:636225.
2. Munoz J, Rodière M, Jeremiah N, et al. Stimulator of interferon genes-associated vasculopathy with onset in infancy: a mimic of childhood granulomatosis with polyangiitis. JAMA Dermatol. 2015;151(8):872–877.
3. Liu Y, Jesus AA, Marrero B, et al. Activated STING in a vascular and pulmonary syndrome. N Engl J Med. 2014;371(6):507–518.
4. ClinVar, unpublished
5. König N, Fiehn C, Wolf C, et al. Familial chilblain lupus due to a gain-of-function mutation in STING. Ann Rheum Dis. 2017;76(2):468–472.
6. Zampeli E, Vakrakou AG, Germenis AE, et al. A case of sting-associated vasculopathy with onset in infancy (SAVI) in a young adult male with a novel TMEM173 gene mutation [abstract]. Ann Rheum Dis. 2017;76(Suppl 2):1132.
7. Melki I, Rose Y, Uggenti C, et al. Disease-associated mutations identify a novel region in human STING necessary for the control of type I interferon signaling. J Allergy Clin Immunol. 2017;140(2):543–552.e5.
8. Keskitalo S, Haapaniemi E, Einarsdottir E, et al. Novel TMEM173 mutation and the role of disease modifying alleles. Front Immunol. 2019;10:2770.
9. Seo J, Kang JA, Suh DI, et al. Tofacitinib relieves symptoms of stimulator of interferon genes (STING)-associated vasculopathy with onset in infancy caused by 2 de novo variants in TMEM173. J Allergy Clin Immunol. 2017;139(4):1396–1399.e12.
10. Lin B, Berard R, Al Rasheed A, et al. A novel STING1 variant causes a recessive form of STING-associated vasculopathy with onset in infancy (SAVI). J Allergy Clin Immunol. 2020;146(5):1204–1208.e6.
11. Konno H, Chinn IK, Hong D, et al. Pro-inflammation associated with a gain-of-function mutation (R284S) in the innate immune sensor STING. Cell Rep. 2018;23(4):1112–1123.
12. Jin L, Xu LG, Yang IV, et al. Identification and characterization of a loss-of-function human MPYS variant. Genes Immun. 2011;12(4):263–269.
13. Yi G, Brendel VP, Shu C, Li P, Palanathan S, Cheng Kao C. Single nucleotide polymorphisms of human STING can affect innate immune response to cyclic dinucleotides. PLoS One. 2013;8(10):e77846.
