# Supplementary material for: Adult-onset STING-associated vasculopathy
Source: J Hum Immun. 2026 May 11;2(4):e20250235. doi: 10.70962/jhi.20250235 (PMC13159526; doi:10.70962/jhi.20250235)
Supplement: Table S2 — shows patient characteristics with reported pathogenic STING1 variants. [file jhi_20250235_tables2.docx]

| **Case** | ***STING1* variant** | **Age of onset  (yrs)** | **Age at last follow-up (yrs)** | **Manifestations** | **Autoimmune diagnosis** | **Other medical  history** | **Current  treatment** | **Prior  treatments** |
| --- | --- | --- | --- | --- | --- | --- | --- | --- |
| 1 | c.842G>A: R281Q | 19 | 44 | inflammatory arthritis, malar rash, serositis, ear chondritis, nasal chondritis, tracheitis, purpuric rashes with cutaneous ulcers,  + ANA, + dsDNA | relapsing polychondritis, SLE | deep vein thrombosis, recurrent sinusitis, pneumonia, recurrent UTI recurrent diverticulitis, cholecystitis, intrabdominal abscess | IVIG,  methylprednisolone | hydroxychloroquine, methotrexate, mycophenolate mofetil, azathioprine, dapsone, cyclophosphamide |
| 2 | c.842G>A: R281Q | NA | 59 | none | none | ocular herpes zoster, type 2 diabetes, migraines, osteoarthritis | none | none |
| 3 | c.842G>A: R281Q | NA | 67 | none | none | HIV , CHF, T2DM | none | none |
| 4 | c.463 G>A: V155M | 30 | 45 | interstitial lung disease, inflammatory arthritis, Raynaud's, +ANA, +RF | RA-ILD | viral meningitis, trigeminal neuralgia | prednisone, azathioprine | mycophenolate mofetil, methotrexate |
| 5 | c.463 G>A: V155M | 30 | 47 | demyelinating CNS lesions | multiple sclerosis | culture-negative septic shock; trigeminal neuralgia | ocrelizumab | dimethyl fumarate |
| 6 | c.463 G>A: V155M | NA | 24 | none | none | preeclampsia, migraines | none | none |

**Supplemental Table 2.** Patient characteristics with reported pathogenic *STING1* variants.
